# Autologous arteriovenous fistula is associated with superior outcomes in elderly hemodialysis patients

**DOI:** 10.1186/s12882-018-1109-9

**Published:** 2018-11-06

**Authors:** Eunjin Bae, Hajeong Lee, Dong Ki Kim, Kook-Hwan Oh, Yon Su Kim, Curie Ahn, Jin Suk Han, Sang-Il Min, Seung-Kee Min, Hyo-Cheol Kim, Kwon Wook Joo

**Affiliations:** 1 Department of Internal Medicine, Gyeongsang National University Changwon Hospital, Changwon, South Korea; 2 Department of Internal Medicine, Seoul National University Hospital, Seoul, South Korea; 3 Kidney Reasearch Institute, Seoul National University College of Medicine, Seoul, South Korea; 4 Department of Surgery, Seoul National University College of Medicine, Seoul, South Korea; 5 Department of Radiology, Seoul National University College of Medicine, Seoul, South Korea; 6 Department of Internal Medicine, Seoul National University College of Medicine, 101 Daehak-Ro, Jongno-Gu, Seoul, 03080 Republic of Korea

**Keywords:** Elderly, Hemodialysis, Vascular access type, Vascular access abandonment, All-cause mortality

## Abstract

**Background:**

The number of elderly patients with end-stage renal disease is increasing rapidly. The higher prevalence of comorbidities and shorter life expectancy in these patients make it difficult to decide on the type of vascular access (VA). We explored the optimal choice for VA in elderly hemodialysis patients.

**Methods:**

We included elderly patients (> 65 years) visiting our VA clinic and divided them into three groups as follows: radiocephalic arteriovenous fistula (AVF), brachiocephalic AVF, and prosthetic arteriovenous graft (AVG). The primary outcomes were VA abandonment and all-cause mortality. The secondary outcome was maturation failure (MF).

**Results:**

Of 529 patients, 61.2% were men. The mean age was 73.6 ± 6.0 years. The VA types were as follows: 49.9% radiocephalic AVF, 31.8% brachiocephalic AVF, and 18.3% AVG. Patients with an AVG tended to be older, female, and have a lower body mass index. More than half of patients (*n* = 302, 57.1%) started dialysis with central catheters, but the proportion of predialysis central catheter placement was not different among the VA types. Radiocephalic AVF was significantly superior to AVG in terms of VA abandonment (*P* = 0.005) and all-cause mortality (*P* < 0.001) in spite of a higher probability of MF. Brachiocephalic AVF was associated with a shorter time to the first needling and fewer interventions before maturation than radiocephalic AVF.

**Conclusions:**

Autologous AVF was suggested as the preferred VA choice in terms of long-term outcomes in elderly patients.

## Background

The number of aging patients with end-stage renal disease (ESRD) is rapidly increasing. According to the United States Renal Data System report, the prevalence of ESRD increased from 4156 per million in 2000 to 6223 per million in 2015 in people aged > 65 years [1]. Similarly, the proportion of elderly ESRD patients aged > 65 years has increased over time, reaching 45.9% in 2016 in Korea [2, 3]. Dialysis initiation in elderly patients with a higher burden of age-related problems is associated with a variety of concerns, including the selection of vascular access (VA).

The current Kidney Disease Outcomes Quality Initiative guidelines support the Fistula First Initiative for all HD patients. Autologous arteriovenous fistulas (AVFs) have been preferred VA for the past decade [4–6] because AVFs have the lowest risk of infectious complications, the longest patency, and superior survival rate despite difficulties with maturation. However, the optimal VA strategy in elderly dialysis patients remains unclear because of their relatively shorter life expectancy, higher prevalence of comorbidities, and difficulty in VA maturation. In recent years, some studies have presented different opinions on the Fistula First Initiative in elderly patients. Some studies have suggested that insertion of an AVG in the pre-dialysis period could be beneficial in elderly patients by sparing transient catheter insertion and related complications [7–9]. Another study suggested a catheter as the main form of dialysis access in very elderly patients needing dialysis in terms of maturation failure (MF) [10]. However, there are concerns about catheter-related bloodstream infections and shorter survival. In elderly patients, few studies have compared the survival rates following AVF and AVG creation for VA. Some studies showed longer survival for AVFs [11–14], whereas other studies showed similar or shorter survival for AVFs compared with AVGs [15–17].

Most previous studies compared one outcome, such as MF, VA abandonment, or patient survival rate, between patients receiving an AVF or AVG rather than comparing the different VA subtypes. With this in mind, the aim of this study was to evaluate which VA type is better for each clinical outcome in elderly Koreans.

## Methods

### Study population

We retrospectively enrolled outpatients visiting Seoul National University Hospital Vascular Access Clinic between January 2008 and March 2014 [18].

Elderly patients aged > 65 years who were maintained on HD were included. Patients who 1) had no VA, 2) visited our clinic only during an emergency, or 3) had undergone intervention or surgical treatment for VA within the last month were excluded. After exclusion, we stratified the remaining patients into three groups according to VA types, as follows: radiocephalic (RC) AVF, brachiocephalic (BC) AVF, and AVG.

### Clinical data collection

We retrospectively reviewed the demographic and clinical data. Body mass index (BMI) was calculated as weight in kg divided by height in m^2^. Laboratory data and etiology of ESRDwere obtained at the time of VA creation. We gathered information from these pre-operative surveillance techniques. We also examined VA duplex ultra-sonography (DUS) findings at the time of the first visit. After surgery for VA creation, we regularly followed-up VA maturation status with a 2–4-week interval until the VA had matured sufficiently. We collected data on the time to the first VA use and whether patients received percutaneous transluminal angioplasty (PTA) due to poor maturation of the VA.

### Outcome assessment

The primary endpoints were VA abandonment and all-cause mortality. VA abandonment was defined as an access that could no longer be used for 1- or 2-needle dialysis as it might be unable to provide adequate flow and/or be deemed unsafe for the patient if the associated problem could not be corrected by medical, surgical, or radiological interventions or rest [18]. For patients who withdrew from the study, we ascertained the mortality data from both an electronic medical record review and Statistics Korea [19].

The secondary endpoint was MF. MF was defined as a VA that could not be used successfully for dialysis from 90 days following its creation, despite radiological or surgical intervention [20].

### Statistical analysis

Differences among the three groups were analyzed using the chi-square test for categorical variables and the analysis of variance *t*-test for continuous variables. The data are presented as mean ± standard deviation, median with range, or frequency (count and percentage). To explore the association between VA type and primary endpoints, a Kaplan-Meier curve was plotted according to VA types. Survival differences were compared using the log-rank test. To explore the association between VA types and Primary endpoints, multivariate Cox proportional hazards regression analysis using backward stepwise process was applied. Variables that showed a significant association (*P* < 0.10) in univariate analysis or were of considerable theoretical relevance were entered into the multivariate Cox proportional hazards regression models.

To assess the relationship between MF and VA types, we excluded patients who died within 90 days or follow up loss, and performed multivariate logistic regression analysis.

Statistical analyses were performed using SPSS version 21.0 for Windows (SPSS Inc., Chicago, IL, USA). Statistical significance was defined as a *P*-value < 0.05.

## Results

### Baseline patient characteristics

A total of 529 patients were included in the final analysis. Among them, 432 (81.7%) patients received an AVF, including 264 (61.1%) RC and 168 (38.9%) BC fistulas. AVGs were placed in 97 (18.3%) patients. The mean age was 73.6 ± 6.0 years and 61.2% of patients were men. Table 1 compares the baseline characteristics of the three VA groups. Patients receiving an AVG were older and had a lower BMI than those who received an RC AVF but were similar to those who received a BC AVF. Furthermore, their hemoglobin levels were higher but serum uric acid (UA) levels were lower than those of patients with AVFs. Otherwise, there were no significant differences according to VA types with respect to blood pressure, laboratory tests, etiology of ESRD, and co-morbidities.

**Table 1:** Patient characteristics by the vascular access type

	Total (*N* = 529)	RC AVF (*N* = 264)	BC AVF (*N* = 168)	AVG (*N* = 97)	*P*
Age (years)	73.6 ± 6.0	72.9 ± 5.8	74.2 ± 6.0	74.9 ± 6.4	0.007
Men (N, %)	324 (61.2)	176 (66.7)	92 (54.8)	56 (57.7)	0.087
BMI (kg/m^2^)	23.1 ± 3.3	23.5 ± 3.1	22.8 ± 3.5	22.7 ± 3.3	0.043
SBP (mmHg)	130.0 ± 19.5	131.0 ± 18.6	128.7 ± 18.1	129.2 ± 24.0	0.473
DBP (mmHg)	69.3 ± 10.8	69.7 ± 10.7	68.4 ± 10.3	70.0 ± 12.1	0.466
Hemoglobin (g/dL)	10.2 ± 1.4	10.1 ± 1.4	10.0 ± 1.4	10.5 ± 1.4	0.039
Albumin (g/dL)	3.5 ± 0.5	3.5 ± 0.5	3.5 ± 0.6	3.4 ± 0.4	0.126
Total chol. (g/dL)	153.4 ± 39.2	151.7 ± 37.5	153.7 ± 38.8	158.1 ± 44.8	0.428
Calcium (mg/dL)	8.5 ± 0.7	8.5 ± 0.7	8.4 ± 0.7	8.6 ± 0.7	0.222
Phosphorus (mg/dL)	4.2 ± 1.1	4.2 ± 1.1	4.1 ± 1.0	4.1 ± 1.2	0.614
Glucose (mg/dL)	123.7 ± 56.6	126.1 ± 57.4	117.2 ± 51.0	128.7 ± 62.8	0.206
PTH (pg/mL)	168.5 ± 149.1	170.8 ± 149.5	173.4 ± 142.6	147.0 ± 169.6	0.670
Uric Acid (mg/dL)	6.7 ± 2.1	6.8 ± 2.1	6.6 ± 2.4	6.2 ± 1.7	0.031
hs-CRP (mg/dL)	2.4 ± 4.3	2.1 ± 3.8	2.4 ± 4.8	3.1 ± 4.6	0.254
Follow up duration (month)	67.1 ± 44.6	71.0 ± 46.5	66.0 ± 41.3	58.1 ± 43.8	0.048
Etiology of ESRD					0.538
DM (N, %)	247 (45.7)	126 (47.7)	74 (44.0)	42 (43.3)	
HTN (N, %)	40 (7.6)	20 (7.6)	13 (7.7)	7 (7.2)	
GN (N, %)	35 (6.6)	18 (6.8)	8 (4.8)	9 (9.3)	
Others (N, %)	61 (11.3)	22 (8.3)	24 (14.3)	14 (14.4)	
Unknown (N, %)	152 (28.7)	78 (29.5)	49 (29.2)	25 (25.6)	
Comorbidities					
DM (N, %)	304 (57.5)	157 (59.5)	93 (55.4)	54 (55.7)	0.648
HTN (N, %)	419 (79.2)	218 (82.6)	131 (78.0)	70 (72.2)	0.087
CAD (N, %)	120 (22.7)	60 (22.7)	32 (19.0)	28 (28.9)	0.184
PVD (N, %)	26 (4.9)	9 (3.4)	11 (6.5)	6 (6.2)	0.276
CVD (N, %)	106 (20.0)	53 (20.1)	27 (16.1)	26 (26.8)	0.110
CHF (N, %)	77 (14.6)	32 (12.1)	31 (18.5)	14 (14.4)	0.191
Malignancy (N, %)	98 (18.3)	75 (17.2)	23 (23.2)	98 (18.3)	0.161

Values are presented as number (%) or mean ± standard deviation

*AVF* arteriovenous fistula, *AVG* arteriovenous graft, *BMI* body mass index, *BC* brachiocephalic, *CAD* coronary artery disease, *CVD* cerebrovascular disease, *CHF* congestive heart failure, *DBP* diastolic blood pressure, *DM* diabetes mellitus, *ESRD* end stage renal disease, *GN* glomerular nephritis, *hs-CRP* high-sensitivity C-reactive protein, *HTN* hypertension, *PTH* parathyroid hormone, *PVD* peripheral vascular disease, *SBP* systolic blood pressure, *RC* radiocephalic

### Preoperative VA-related characteristics

More than half of patients (*n* = 302, 57.1%) started their HD using a CVC. The mean duration of CVC use was 113.6 ± 73.2 days. Of these, 27 (9.0%) had VA abandonment, 30 (9.9%) died, 57 (22.4%) could not use their VA due to MF, 94 (33%) received PTA and 24 (8.4%) received 2nd or revision operation. Before access creation, 337 (63.7%) patients received preoperative surveillance for artery and vein status. Among them, 207 (61.4%) patients were evaluated by DUS, 60 (17.8%) by venography, and 69 (20.5%) by both DUS and venography. A total of 112 (21.2%) patients received PTA before maturation. The median time to the first use of the VA was 64.0 (14.0–124.0) days.

Table 2 compares the preoperative VA-related clinical characteristics according to VA types. There was no difference in CVC placement proportion and duration according to VA types. Patients who underwent AVG placement tended to receive more aggressive preoperative surveillance, although their maturation time was shorter and proportion of vascular intervention before maturation was lower than in patients who received AVFs. Patients with an RC AVF had the lowest proportion of preoperative vascular surveillance. However, their rate of PTA before maturation was highest and their time to needling was longest among all of the VA types assessed. The proportion of patients with a BC AVF receiving intervention before maturation was much lower than that of patients with an RC AVF but similar to that of patients with an AVG.

**Table 2:** Analysis of the clinical characteristics before first use of vascular access according to vascular access type

		Total (*N* = 529)	RC AVF (*N* = 264)	BC AVF (*N* = 168)	AVG (*N* = 97)	*P*
CVC	None	227 (42.9)	121 (45.8)	69 (41.1)	37 (38.1)	0.358
	IJC	3 (0.6)	2 (0.8)	0 (0)	1 (1.0)	
	Permanent catheter	299 (56.5)	141 (53.4)	99 (58.9)	59 (60.0)	
CVC duration (days)		113.4 ± 73.5	115.3 ± 68.7	112.7 ± 63.7	109.3 ± 101.4	0.891
Preoperative surveillance	None	192 (36.4)	123 (46.8)	53 (31.5)	16 (16.5)	< 0.001
	Duplex ultrasonography	207 (39.2)	102 (38.8)	85 (50.6)	20 (20.6)	
	Venography	60 (11.4)	17 (6.5)	19 (13.3)	24 (24.7)	
	Both	69 (13.1)	21 (8.0)	11 (6.5)	37 (38.1)	
Time to 1st use (days)		64.0 (14.0–124.0)	75.0 (12.0–138.0)	65.5 (8.8–122.3)	35.0 (5.0–65.0)	0.001
PTA before maturation		112 (21.2)	70 (26.5)	27 (16.1)	15 (15.5)	0.011

Values are presented as number (%), mean ± standard deviation, or median with range

*AVF* arteriovenous fistula, *AVG* arteriovenous graft, *BA* brachial artery, *BC* brachiocephalic, *CVC* central vein catheter, *IJC* internal jugular catheter, *PSV* peak systolic velocity, *PTA* percutaneous transluminal angioplasty, *RC* radiocephalic

### DUS findings

Table 3 compares the DUS findings at the time of the first use according to VA types. The diameter of the brachial artery (BA) was not different according to the type of VA. BA flow and peak systolic velocity (PSV) was highest in BC fistulas. In addition, needling-site cephalic venous flow was highest in BC AVFs. PSV of the cephalic vein was highest in AVGs.

**Table 3:** Analysis of the duplex ultrasonography findings before first use of vascular access

		Total (*N* = 529)	RC AVF (*N* = 264)	BC AVF (*N* = 168)	AVG (*N* = 97)	*P*
Duplex ultrasonography	BA diameter (cm)	5.7 ± 3.7	5.7 ± 4.4	5.7 ± 3.5	5.6 ± 1.1	0.955
	BA flow (ml/min)	1009.6 ± 610.3	860.8 ± 477.8	1226.6 ± 743.7	1005.6 ± 535.0	< 0.001
	BA PVS (cm/sec)	207.0 ± 84.7	188.1 ± 68.1	238.9 ± 99.2	195.0 ± 76.8	< 0.001
	Cephalic vein diameter	5.7 ± 6.1	4.9 ± 3.0	6.6 ± 6.7	6.0 ± 10.2	< 0.001
	Cephalic vein flow	911.8 ± 664.8	726.5 ± 505.3	1153.2 ± 788.3	1008.8 ± 672.9	< 0.001
	Cephalic vein PSV	161.7 ± 73.3	146.3 ± 64.6	167.7 ± 72.6	194.2 ± 85.2	0.032

Values are presented as number (%), mean ± standard deviation, or median with range

*AVF* arteriovenous fistula, *AVG* arteriovenous graft, *BA* brachial artery, *BC* brachiocephalic, *PSV* peak systolic velocity, *RC* radiocephalic

### Outcomes according to VA types

**Fig. 1:**
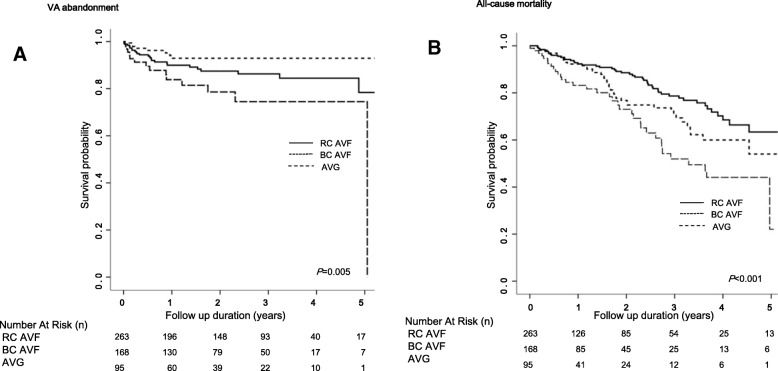
Comparison of primary endpoints by vascular access type. Kaplan-Meier curve for vascular access abandonment (**a**), and all-cause mortality (**b**) according to vascular access type

During a mean follow-up of 66.9 ± 44.5 months, VA abandonment occurred in 8.2% (*n* = 43) and death by any cause occurred in 24.2% (*n* = 128) of elderly dialysis patients. Table 4 presents the clinical outcomes according to VA types. Figure 1 shows the VA abandonment and all-cause mortality rates according to VA types obtained using the Kaplan-Meier method. The VA abandonment rate was highest in AVGs, followed by RC AVFs and BC AVFs (*P* = 0.005). In terms of all-cause mortality, patients with an AVG showed the worst results, followed by those with a BC AVF and RC AVF (*P* < 0.001).

**Table 4:** Clinical outcomes of the elderly hemodialysis patients according to VA Types

	Total (*N* = 302)	RC AVF (*N* = 143)	BC AVF (*N* = 99)	AVG (*N* = 60)	*P*
VA abandonment (n, %)	43 (8.2)	22 (8.4)	8 (4.8)	13 (13.5)	0.043
All-cause mortality (n, %)	44 (8.3)	17 (6.4)	11 (6.5)	16 (16.5)	0.005
Maturation failure (n, %)	136 (33.0)	84 (40.0)	43 (31.6)	9 (13.6)	< 0.001
PTA before maturation (n, %)	112 (21.2)	70 (26.5)	27 (16.1)	15 (15.5)	0.011
Secondary operation (n, %)	30 (6.1)	8 (3.3)	12 (7.7)	10 (11.2)	0.018

Table 5 summarizes the results of multivariate Cox regression analysis for outcomes. In multivariate Cox regression analysis, AVGs significantly elevated the VA abandonment risk (adjusted HR 2.77, 95% CI 1.22–6.27, *P* = 0.033) compared with RC AVFs. Additionally, hemoglobin level (HR 1.41, 95% CI 1.11–1.81, *P* = 0.006) and BA diameter (HR 0.59, 95% CI 0.37–0.93, *P* = 0.024) were significantly associated with VA abandonment. In terms of all-cause mortality, AVGs were an independent risk factor for mortality (adjusted HR 2.65, 95% CI 1.52–4.63, *P* = 0.003). Age (adjusted HR 1.08, 95% CI 1.05–1.12, *P* < 0.001) and peripheral vascular disease (adjusted HR 2.39, 95% CI 1.13–5.06, *P =* 0.023) were significantly associated with all-cause mortality.

**Table 5:** Hazard ratios of primary endpoints in elderly patients

	VA abandonment		All-cause mortality		Maturation failure	
	HR (95% CI)	*P* value	HR (95% CI)	*P* value	OR (95% CI)	*P* value
Age	–	–	1.08 (1.05–1.12)	< 0.001		
Hemoglobin	1.41 (1.11–1.81)	0.006	–	–		
Albumin	–	–	–	–	2.61 (1.6.0–4.25)	< 0.001
VA type (ref. RC AVF)		0.033		0.003		0.010
BC AVF	0.97 (0.35–2.68)	0.955	1.54 (0.92–2.57)	0.102	0.81 (0.47–1.42)	0.464
AVG	2.77 (1.22–6.27)	0.015	2.65 (1.52–4.63)	0.001	0.24 (0.09–0.60)	0.002
PVD	–	–	2.39 (1.13–5.06)	0.023		
BA diameter	0.59 (0.37–0.93)	0.024	–	–		

Adjusted for age, sex, BMI, systolic pressure, Hemoglobin, cholesterol, albumin, calcium, phosphorus, DM, CAD, PVD, CVD, CHF, VA type, history of CVC, duplex U/S findings (Brachial a. diameter, Brachial a. flow, Needling site diameter, Needling site flow)

*VA* vascular access, *BMI* body mass index, *CHF* congestive heart failure, *BA* brachial artery, *SBP* systolic blood pressure, *PVD* peripheral vascular disease, *HR* hazard ratio, *CI* confidence index, *OR* odds ratio

MF was observed in 33.0% (*n* = 136) of patients. The rate was highest for RC AVFs (*n* = 84, 40.0%), followed by BC AVFs (*n* = 43, 31.6%) and AVGs (*n* = 9, 13.3%). When we explored the factors associated with MF, AVGs were associated with significantly lower risks of MF than RC fistulas (adjusted odds ratio 0.24, 95% CI 0.09–0.60, *P* = 0.002). BC fistulas tended to have a lower MF risk than RC fistulas, although this difference was not statistically significant.

### Outcomes according to VA types in very elderly patients

**Fig. 2:**
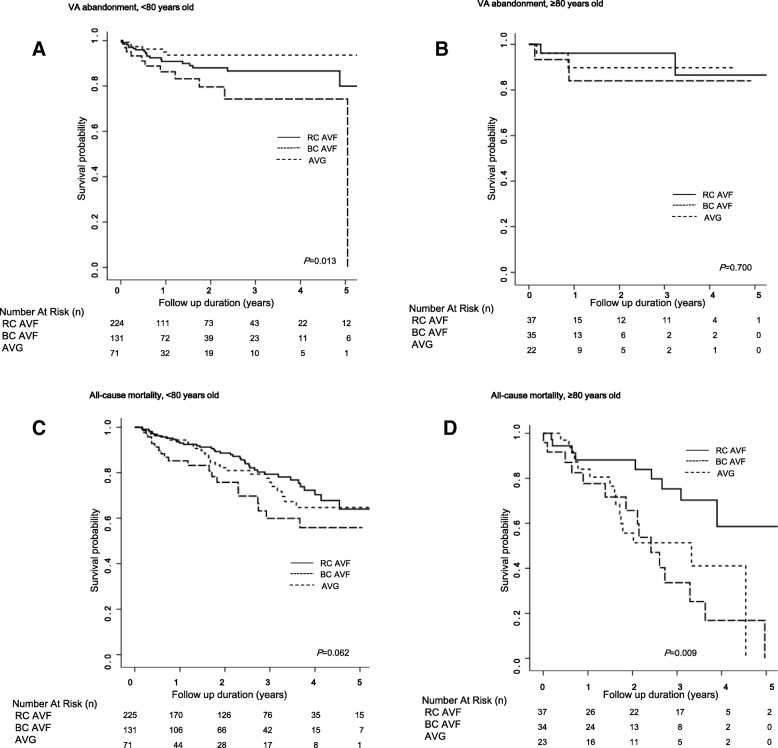
Comparison of primary endpoints according to vascular access type in very elderly patients. Kaplan-Meier curve for vascular access abandonment in age < 80 years old (**a**), ≥80 years old (**b**) and all-cause mortality in age < 80 years old (**c**), ≥80 years old (**d**) according to vascular access type

We identified the outcomes associated with different VA types in very elderly patients (≥80 years). Figure 2b shows the VA abandonment rate in patients aged > 80 years determined using the Kaplan Meier method. There was no statistically significant relationship and no inferiority of AVFs compared to AVGs. In addition, AVFs were significantly superior to AVGs in terms of all-cause mortality. Table 6 shows the effect of VA type on outcomes in patients aged > 80 years. In very elderly patients, RC AVFs were associated with lower risks of all-cause mortality than BC AVFs and AVGs. VA types did not have a significant effect on other outcomes, such as VA abandonment and MF.

**Table 6:** Hazard ratios of vascular access type on endpoints according to age

	VA abandonment		All-cause mortality		Maturation failure	
	HR (95% CI)	*P* value	HR (95% CI)	*P* value	OR (95% CI)	*P* value
Age < 80 years						
VA type (ref. RC AVF)		0.019				0.015
BC AVF	0.55 (0.18–1.70)	0.299			0.68 (0.39–1.20)	0.184
AVG	2.71 (1.09–6.73)	0.032			0.23 (0.09–0.65)	0.005
Age ≥ 80 years						
VA type (ref. RC AVF)				0.002		
BC AVF			3.47 (1.34–9.01)	0.011		
AVG			6.30 (2.29–17.35)	< 0.001		

Adjusted for age, sex, BMI, systolic blood pressure, Hemoglobin, cholesterol, albumin, calcium, phosphorus, DM, CAD, PVD, CVD, CHF, VA type, history of CVC, duplex U/S findings (BA diameter, BA flow, Cephalic vein diameter, Cephalic vein flow)

*AVF* arteriovenous fistula, *AVG* arteriovenous graft, *BA* brachial artery, *BMI* body mass index, *BC* brachiocephalic, *CAD* coronary artery disease, *CVC* central vein catheter, *CVD* cerebrovascular disease, *CHF* congestive heart failure, *DM* diabetes mellitus, *PVD* peripheral vascular disease, *PSV* peak systolic velocity, *PTA* percutaneous transluminal angioplasty, *RC* radiocephalic, *U/S* ultrasonography, *HR* hazard ratio, *CI* confidence index, *OR* odds ratio

## Discussion

In this study, we investigated baseline characteristics, DUS findings, and outcomes according VA types in elderly HD patients. Our aim was to determine the optimal VA type in elderly patients. We found that AVFs were superior to AVGs with respect to all-cause mortality and VA abandonment, although AVFs were associated with a higher risk of MF in elderly HD patients. Among AVFs, BC fistulas showed similar benefits to RC fistulas in terms of VA abandonment, all-cause mortality, and MF risk. However, BC fistulas had a lower intervention rate than RC fistulas. Moreover, DUS findings were more favorable for BC fistulas than RC fistulas. Consequently, BC fistulas might be a VA type that is not inferior to RC fistulas for elderly dialysis patients.

Inadequate VA leads to recurrent PTA, re-operation, and CVC insertion, which increase the risk of infection and mortality. In addition, inadequate VA is related to poor quality of life. Researchers have investigated various aspects of VA, such as timing, placement, and type. Some previous studies compared RC and BC AVFs. In terms of patency, BC AVFs have advantages over RC AVFs [7, 13, 21–23], whereas BC AVFs are associated with more steal syndrome than RC AVFs [24]. The present study demonstrated that AVFs are superior to AVGs in terms of all-cause mortality and VA abandonment but not MF. In patients aged 65–80 years, BC AVFs showed no significant difference in all-cause mortality compared to RC AVFs and favorable outcomes in terms of VA abandonment and MF. In patients aged > 80 years, BC AVFs showed inferior outcomes to RC AVFs in terms of all-cause mortality. BC AVFs were associated with less PTA before maturation and better DUS findings than RC AVFs. In view of these findings, AVFs should be considered as the first-choice VA rather than AVGs in elderly patients. Furthermore, it is not necessary to insist on RC AVFs. Rather, the choice between BC and RC AVFs should be determined based on blood vessel status.

In this study, RC AVFs accounted for the largest proportion of AVFs at 61.1% in elderly patients, showing a large proportion of RC AVFs were placed compared to other studies. Other previous studies showed that 24.7% to 60.7% patients received RC AVFs in AVFs [13, 21–23]. It might be following reasons; skilled surgical technique, recently enrolled patients could have better vascular condition than the patients in the previous studies. There were no significant differences in gender or age between our study and previous studies.

The results of this study should be interpreted with caution. Although all patients were elderly, patients with RC AVFs had better vascular status and fewer co-morbidities than patients with other BC AVFs or AVGs. In this study, patients in the RC AVF group were the youngest and their BMI and UA level were higher than those of patients in the other VA type groups. The higher BMI and UA level could reflect the good nutritional status of patients in the RC AVF group in this study. Although, we adjusted for nutritional status and co-morbidities, the relationship between all-cause mortality and RC AVF should be interpreted as a surrogate marker rather than as an effect of RC AVF itself.

The present study investigated details related to VA in elderly dialysis patients, such as methods of VA surveillance before the first dialysis, DUS findings, interventions, first dialysis methods, MF, and VA abandonment. Previous studies mainly focused on outcome-related factors, whereas this study evaluated the overall characteristics associated with VA, such as the process of creating a VA and outcomes during the follow-up period.

We analyzed DUS findings, which were associated with the clinical outcomes of VA creation. Among the DUS findings, only BA diameter was significantly associated with VA abandonment. One previous study [25] showed that BA diameter was positively correlated with AVF success. Other studies [26, 27] reported good BA flow rate consequent to RC wrist AVF maturation. As such, the BA represents an ideal site for studying distal AVFs. As yet, there is no definite DUS finding that can predict VA outcomes. However, the results of this study could represent evidence that the BA is relatively important in DUS findings, especially in elderly patients.

The present study had some limitations. First, the study was retrospective in nature. As such, it was difficult to infer causal relationships and selection bias cannot be completely ruled out. Second, since most of the study population was Asian, the data cannot be generalized to other races. Third, DUS was performed by well-trained specialists but not by the same person, which could have led to differences in the DUS results. To overcome these limitations, well-planned prospective, multicenter studies are needed.

## Conclusions

In conclusion, the fistula first strategy could also be applied to elderly HD patients with respect to VA abandonment and all-cause mortality. BC AVFs could be considered as the first-choice VA depending on the patient’s condition.

## Abbreviations

AVF: Autologous arteriovenous fistula
AVG: Arteriovenous graft
BA: Brachial artery
BC: Brachiocephalic
BMI: Body mass index
CI: Confidence interval
CVC: Central venous catheter
DUS: Duplex ultra-sonography
ESRD: End-stage renal disease
HD: Hemodialysis
HR: hazard ratio
PSV: Peak systolic velocity
PTA: Percutaneous transluminal angioplasty
RC: Radiocephalic
VA: Vascular access
